# Supplementary material for: Machine Learning Accelerates Crystallization for Structure Determination
Source: Angew Chem Int Ed Engl. 2026 May 4;65(25):e1218503. doi: 10.1002/anie.1218503 (PMC13266958; doi:10.1002/anie.1218503)

checkCIF/PLATON report

Structure factors have been supplied for datablock(s) lcz047

THIS REPORT IS FOR GUIDANCE ONLY. IF USED AS PART OF A REVIEW PROCEDURE FOR PUBLICATION, IT SHOULD NOT REPLACE THE EXPERTISE OF AN EXPERIENCED CRYSTALLOGRAPHIC REFEREE.

No syntax errors found. CIF dictionary Interpreting this report

Datablock: lcz047

Bond precision:	C-C = 0.0118 A		Wavelength=1.54184
Cell:	a=11.8873(3)	b=12.7795(5)	c=23.8506(6)
	alpha=90	beta=91.767(3)	gamma=90
Temperature:	100 K		

	Calculated	Reported
Volume	3621.51(19)	3621.51(19)
Space group	P 21/n	P 1 21/n 1
Hall group	-P 2yn	-P 2yn
Moiety formula	C30 H17 Ag3 F18 N6 O3	C15 H3 Ag3 F18 N6, C15 H14 O3
Sum formula	C30 H17 Ag3 F18 N6 O3	C30 H17 Ag3 F18 N6 O3
Mr	1175.11	1175.10
Dx,g cm-3	2.155	2.155
Z	4	4
Mu (mm-1)	14.162	14.162
F000	2264.0	2264.0
F000'	2274.73	
h,k,lmax	15,16,30	15,15,30
Nref	7775	7395
Tmin,Tmax	0.147,0.059	0.190,1.000
Tmin'	0.024	

Correction method= # Reported T Limits: Tmin=0.190 Tmax=1.000
AbsCorr = MULTI-SCAN

```
Data completeness= 0.951            Theta(max)= 78.316

                                                        wR2(reflections)=
R(reflections)= 0.0637( 6336)                           0.1861( 7395)

S = 1.089                 Npar= 543
```

The following ALERTS were generated. Each ALERT has the format
test-name_ALERT_alert-type_alert-level.
Click on the hyperlinks for more details of the test.

Alert level C

```
PLAT042_ALERT_1_C Calc. and Reported MoietyFormula Strings  Differ     Please Check
              Calc: C30 H17 Ag3 F18 N6 O3
              Rep.: C15 H3 Ag3 F18 N6, C15 H14 O3
PLAT342_ALERT_3_C Low Bond Precision on  C-C Bonds ..............    0.01181 Ang.
PLAT906_ALERT_3_C Large K Value in the Analysis of Variance ......      4.579 Check
PLAT911_ALERT_3_C Missing FCF Refl Between Thmin & STh/L=    0.600         30 Report
                0  2  0,  13  4  0,  12  8  0,  -1  0  1,   1  0  1,   1  2  1,
              -13  6  1, -12  8  1,  12  8  1, -10  0  4,  12  6  4,   6  8  4,
               -1  7  6,   0  7  6,  -2  8  6, -12  6  7,  -3  6  8,   6  7  8,
               13  3  9,  -2  6 10,  12  3 11,  11  0 13,  -1  8 13,   8 10 14,
                3  3 15,   3  0 19,   2  9 19,   0  0 20,  -8  0 22,  -1  5 27,
PLAT934_ALERT_3_C Number of (Iobs-Icalc)/Sigma(W) > 10 Outliers ..          1 Check
               13  7  3,
PLAT971_ALERT_2_C Check Calcd Resid. Dens.  1.19Ang From Ag03          1.95 eA-3
PLAT971_ALERT_2_C Check Calcd Resid. Dens.  1.77Ang From F00T          1.83 eA-3
PLAT971_ALERT_2_C Check Calcd Resid. Dens.  1.10Ang From Ag03          1.75 eA-3
PLAT971_ALERT_2_C Check Calcd Resid. Dens.  0.95Ang From N009          1.63 eA-3
PLAT971_ALERT_2_C Check Calcd Resid. Dens.  1.13Ang From Ag02          1.54 eA-3
PLAT972_ALERT_2_C Check Calcd Resid. Dens.  1.71Ang From Ag03         -1.55 eA-3
```

Alert level G

```
PLAT083_ALERT_2_G SHELXL Second Parameter in WGHT  Unusually Large      39.33 Why ?
PLAT242_ALERT_2_G Low    'MainMol' Ueq as Compared to Neighbors of       C012 Check
PLAT242_ALERT_2_G Low    'MainMol' Ueq as Compared to Neighbors of       C01H Check
PLAT242_ALERT_2_G Low    'MainMol' Ueq as Compared to Neighbors of       C01J Check
PLAT242_ALERT_2_G Low    'MainMol' Ueq as Compared to Neighbors of       C01K Check
PLAT242_ALERT_2_G Low    'MainMol' Ueq as Compared to Neighbors of       C01L Check
PLAT242_ALERT_2_G Low    'MainMol' Ueq as Compared to Neighbors of       C01M Check
PLAT434_ALERT_2_G Short Inter HL..HL Contact F00K     ..F00L     .       2.72 Ang.
                                      1/2-x,-1/2+y,3/2-z  =      2_546 Check
PLAT434_ALERT_2_G Short Inter HL..HL Contact F00N     ..F00N     .       2.66 Ang.
                                              -x,2-y,1-z  =      3_576 Check
PLAT720_ALERT_4_G Number of Unusual/Non-Standard Labels .........         77 Note
              Ag01    Ag02    Ag03    F004    F005    F006    F007    F008
              N009    F00A    F00B    N00C    F00D    F00E    F00F    F00G
              N00H    N00I    O00J    F00K    F00L    O00M    F00N    N00O
              N00P    F00Q    F00R    O00S    F00T    F00U    C00V    H00V
              C00W    C00X    C00Y    H00Y    C00Z    C010    C011    C012
              C013    H013    C014    H014    C015    C016    C017    H017
              C018    C019    C01A    C01B    C01C    H01C    C01D    H01D
              C01E    H01E    C01F    H01F    C01G    H01G    C01H    C01I
```

```
              H01I    C01J    C01K    C01L    C01M    C01N    H01A    H01B
              H01H    C01O    H01J    H01K    H01L
PLAT883_ALERT_1_G Absent Datum for _atom_sites_solution_primary ..     Please Do !
PLAT912_ALERT_4_G Missing # of FCF Reflections Above STh/L=  0.600        327 Note
PLAT941_ALERT_3_G Average HKL Measurement Multiplicity ..........         2.7 Low
PLAT969_ALERT_5_G The 'Henn et al.' R-Factor-gap value ..........       5.474 Note
              Predicted wR2: Based on SigI**2  3.40 or SHELX Weight 17.10
PLAT978_ALERT_2_G Number C-C Bonds with Positive Residual Density.          0 Info
```

```
   0 ALERT level A = Most likely a serious problem - resolve or explain
   0 ALERT level B = A potentially serious problem, consider carefully
  11 ALERT level C = Check. Ensure it is not caused by an omission or oversight
  15 ALERT level G = General information/check it is not something unexpected

   2 ALERT type 1 CIF construction/syntax error, inconsistent or missing data
  16 ALERT type 2 Indicator that the structure model may be wrong or deficient
   5 ALERT type 3 Indicator that the structure quality may be low
   2 ALERT type 4 Improvement, methodology, query or suggestion
   1 ALERT type 5 Informative message, check
```

Validation response form

Please find below a validation response form (VRF) that can be filled in and pasted into your CIF.

```
# start Validation Reply Form
_vrf_PLAT042_lcz047
;
PROBLEM: Calc. and Reported MoietyFormula Strings  Differ     Please Check
RESPONSE: ...
;
_vrf_PLAT342_lcz047
;
PROBLEM: Low Bond Precision on  C-C Bonds ..............    0.01181 Ang.
RESPONSE: ...
;
_vrf_PLAT906_lcz047
;
PROBLEM: Large K Value in the Analysis of Variance ......      4.579 Check
RESPONSE: ...
;
_vrf_PLAT911_lcz047
;
PROBLEM: Missing FCF Refl Between Thmin & STh/L=    0.600         30 Report
RESPONSE: ...
;
_vrf_PLAT934_lcz047
;
PROBLEM: Number of (Iobs-Icalc)/Sigma(W) > 10 Outliers ..          1 Check
RESPONSE: ...
;
_vrf_PLAT971_lcz047
;
PROBLEM: Check Calcd Resid. Dens.  1.19Ang From Ag03           1.95 eA-3
RESPONSE: ...
;
```

```
_vrf_PLAT972_lcz047
;
PROBLEM: Check Calcd Resid. Dens.  1.71Ang From Ag03           -1.55 eA-3
RESPONSE: ...
;
# end Validation Reply Form
```

It is advisable to attempt to resolve as many as possible of the alerts in all categories. Often the minor alerts point to easily fixed oversights, errors and omissions in your CIF or refinement strategy, so attention to these fine details can be worthwhile. It is up to the individual to critically assess their own results and, if necessary, seek expert advice.

PLATON version of 17/09/2025; check.def file version of 17/09/2025

---

duplicate check

No duplication found

Datablock lcz047 - ellipsoid plot

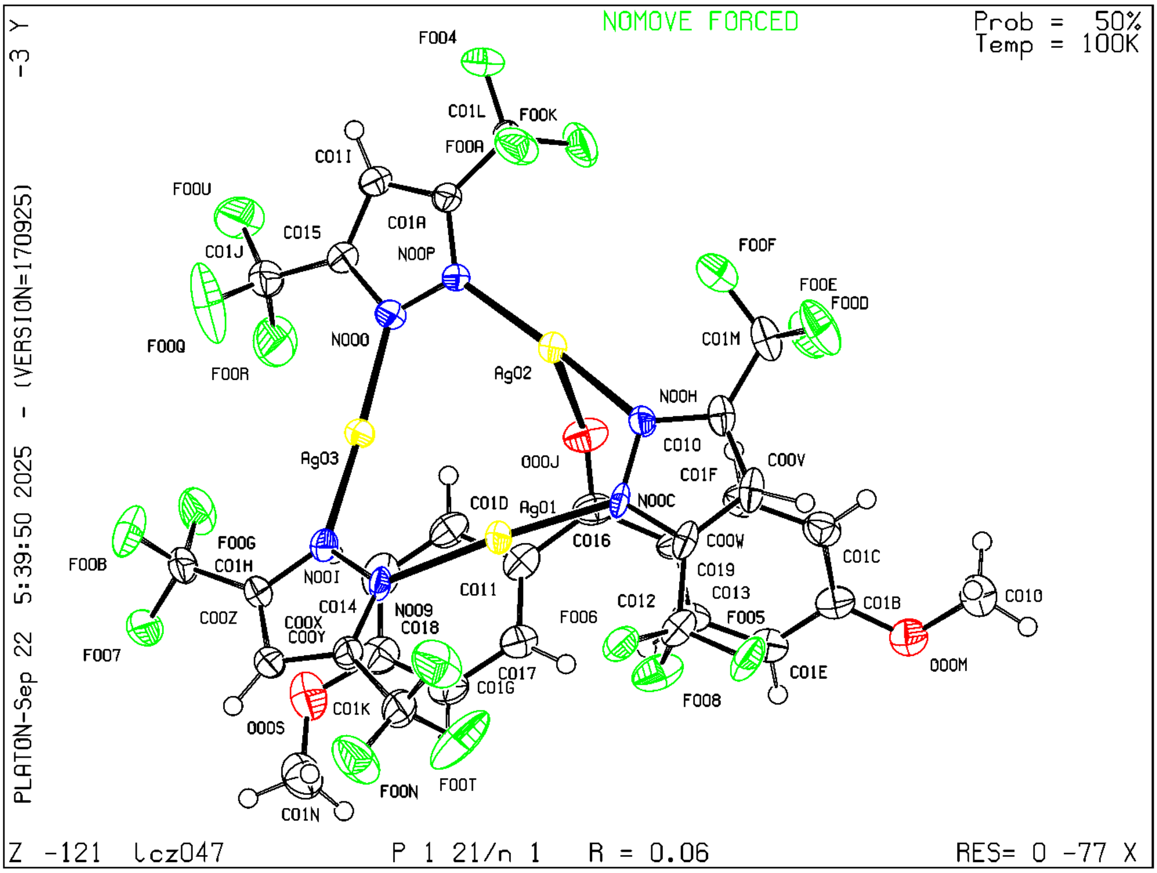

Supplement: Supplementary file 2 — Supporting File 2: anie72462‐sup‐0002‐DataFile.zip. [file ANIE-65-e1218503-s002.zip › supplementary_materials_crystallographic_data submit.zip!/cif_and_checkcif(1-114)/Ag3Pz3_47/Ag3Pz3_47_checkcif.pdf]
